# Expression and Characterization of a Novel Glycerophosphodiester Phosphodiesterase from *Pyrococcus furiosus* DSM 3638 That Possesses Lysophospholipase D Activity

**DOI:** 10.3390/ijms17060831

**Published:** 2016-05-30

**Authors:** Fanghua Wang, Linhui Lai, Yanhua Liu, Bo Yang, Yonghua Wang

**Affiliations:** 1 School of Food Science and Engineering, South China University of Technology, Guangzhou 510640, China; sdzbwfh@126.com; 2 School of Bioscience and Bioengineering, South China University of Technology, Guangzhou 510006, China; llhhit@163.com (L.L.); yhlscut@163.com (Y.L.); yangbo@scut.edu.cn (B.Y.)

**Keywords:** glycerophosphodiester phosphodiesterase, *Pyrococcus furiosus* DSM 3638, biochemical properties, lysophospholipase D activity, substrate selectivity

## Abstract

Glycerophosphodiester phosphodiesterases (GDPD) are enzymes which degrade various glycerophosphodiesters to produce glycerol-3-phosphate and the corresponding alcohol moiety. Apart from this, a very interesting finding is that this enzyme could be used in the degradation of toxic organophosphorus esters, which has resulted in much attention on the biochemical and application research of GDPDs. In the present study, a novel GDPD from *Pyrococcus furiosus* DSM 3638 (pfGDPD) was successfully expressed in *Escherichia coli* and biochemically characterized. This enzyme hydrolyzed bis(*p*-nitrophenyl) phosphate, one substrate analogue of organophosphorus diester, with an optimal reaction temperature 55 °C and pH 8.5. The activity of pfGDPD was strongly dependent on existing of bivalent cations. It was strongly stimulated by Mn^2+^ ions, next was Co^2+^ and Ni^2+^ ions. Further investigations were conducted on its substrate selectivity towards different phospholipids. The results indicated that except of glycerophosphorylcholine (GPC), this enzyme also possessed lysophospholipase D activity toward both *sn*1-lysophosphatidylcholine (1-LPC) and *sn*2-lysophosphatidylcholine (2-LPC). Higher activity was found for 1-LPC than 2-LPC; however, no hydrolytic activity was found for phosphatidylcholine (PC). Molecular docking based on the 3D-modeled structure of pfGDPD was conducted in order to provide a structural foundation for the substrate selectivity.

## 1. Introduction

Glycerophosphodiesterase phosphodiesterases (GDPDs; EC 3.1.4.46) are ubiquitous among prokaryotic and eukaryotic organisms. The primary function of these enzymes is the degradation of various glycerophosphodiesters to glycerol-3-phosphate (G3P) and corresponding alcohol moiety (e.g., choline, serine, ethanolamine, inositol). It is a key enzyme in the phospholipid metabolism pathway which maintains G3P concentrations, for phospholipid remodelling and synthesis [1]. GDPDs have been characterized from bacterial, yeast, plant and mammalian cells [2]. In *Escherichia coli*, two enzymes are found: the periplasmic (GlpQ) and cytosolic (UgpQ) GDPDs, and these enable the bacterium to use GDPDs to provide G3P for lipid biosynthesis [3,4]. Apart from this, some GDPDs originating from pathogenic bacteria, such as *Haemophilus influenza* [5] and *Mycoplasma pneumonia* [6], have been identified as virulence factors. So far, seven mammalian homologs of bacterial GDPDs have been identified (GDE1-GDE7), six of which were membrane proteins [7]. The proposed roles for these mammalian GDPDs include osteoblast and neuron differentiation, skeletal muscle development, anandamine biosynthesis and osmolyte regulation [8,9,10,11,12]. Recently, a glycerophosphodiesterase (GpdQ) from *Enterobacter aerogenes* has been proposed in the bioremediation of organophosphorus esters (OP). It has been suggested that GpdQ might be part of a catabolic pathway for the degradation of phosphotriesters [13] or might be capable of hydrolyzing a close analogue of EA 2192, which is the most toxic and persistent degradation product of the nerve agent VX [14]. Therefore, screening and characterization of novel GDPDs that have great potential in the bioremediation applications has caused much interest amongst scientists.

In this study, a novel GDPD from *Pyrococcus furiosus* DSM 3638 (pfGDPD) was successfully expressed and we extensively characterized the pfGDPD protein, including its phylogenetic relationship, 3D model and enzyme characteristics. pfGDPD was found to have hydrolytic activity toward non-physiological substrate bis(*p*-nitrophenyl) phosphate (BpNPP), an important OP alternate that is commonly used for screening of organophosphate degrading enzymes with higher activity. Finally, hydrolytic reactions were performed and we found for the first time that pfGDPD showed phospholipase D activity towards l-α-glycerophosphorylcholine (GPC), and also showed lysophospholipase D activity toward l-α-lysophosphatidylcholine (lyso-PC). These data not only contribute to our current understanding of GDPDs and of their physiological roles via the control of GPC and lyso-PC metabolism in cells but also expand the application potential of its utility in bioremediation.

## 2. Results

### 2.1. Sequence Analysis of pfGDPD

**Figure 1:**
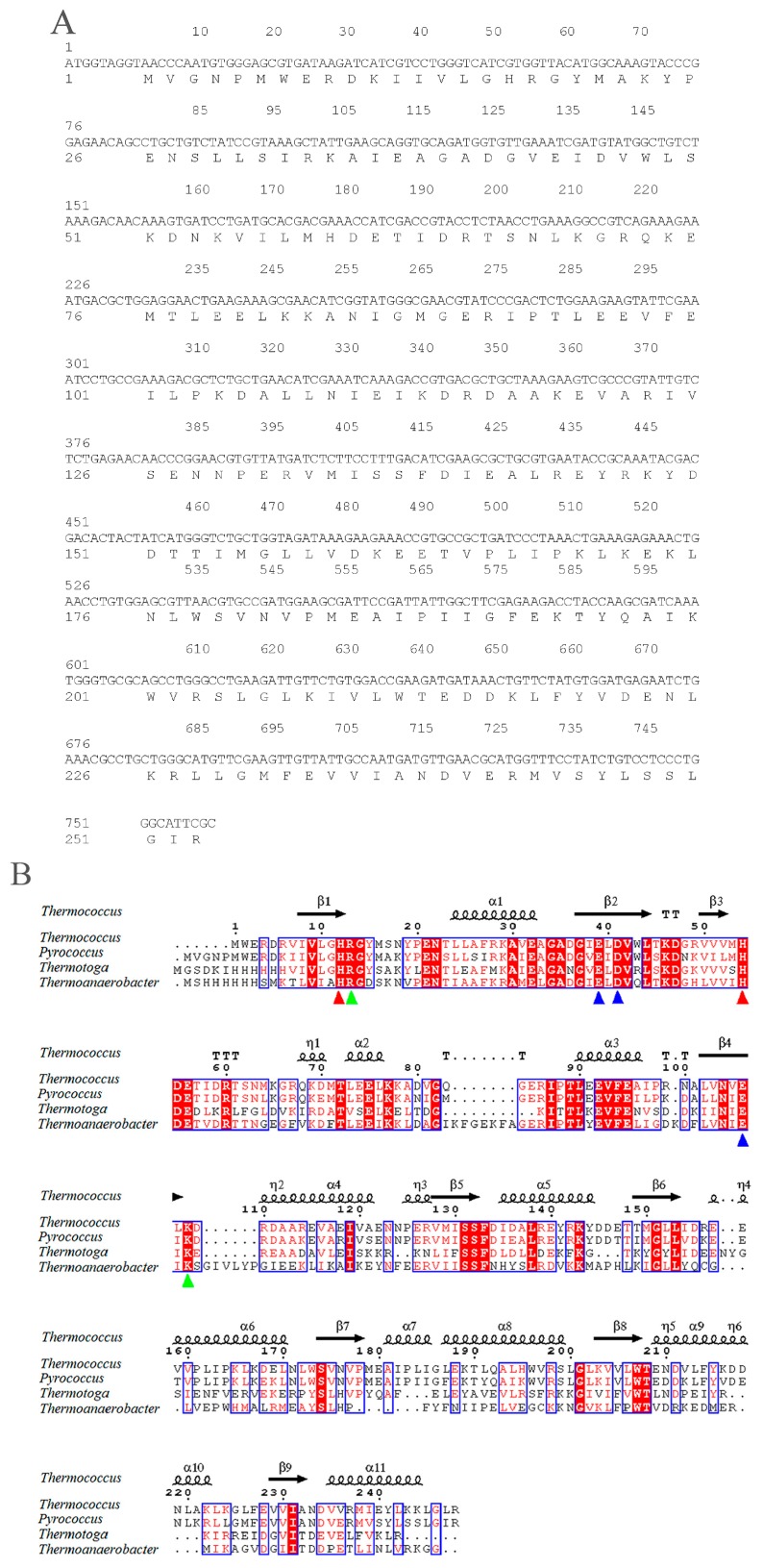
Information on the synthetic sequence of glycerophosphodiester phosphodiesterases from *Pyrococcus furiosus* DSM 3638 (pfGDPD) (**A**) and multiple sequence alignment of pfGDPD amino acid sequence with those of known GDPD proteins available in Protein Data Bank (PDB); (**B**) Identical residues were marked with a red background, and the highly conserved residues were showed in red font. Secondary structures of GDPD are shown above the alignments. The catalytic triads of two histidines are indicated with red triangles. Triangles in green indicate strictly conserved residues in GDPDs. Triangles in blue indicated the divalent cation-binding related residues in that cleft of GDPDs. GDPD from *Thermococcus kodakarensis* KOD1(PDB: 4OEC); GDPD from *Thermotoga maritima* (PDB: 1O1Z); GDPD from *Thermoanaerobacter tengcongensis* (PDB: 2PZ0) were used for alignment.

**Figure 2:**
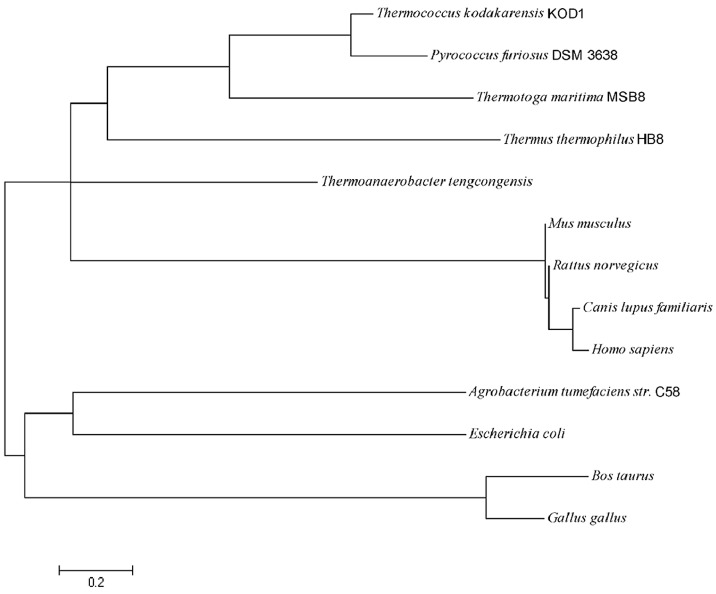
Phylogenetic tree of the known GDPDs. Phylogenetic analysis with Neighbor-joining (NJ) was conducted using MEGA 6.0 software (Hachioji, Tokyo, Japan). GDPD protein sequences that were used for this analysis are XP_536954.2 (*Canis lupus familiaris*), NP_116004 (*Rattus norvegicus*), NP_001032348.1 (*Gallus gallus*), NP_001094611.1 (*Bos taurus*), NP_534690.1 (*Agrobacterium tumefaciens* str. C58), EFJ64212 (*Escherichia coli*), NP_057725 (*Homo sapiens*). NP_062526 (*Mus musculus*), WP_004082090 (*Thermotoga maritima* MSB8), WP_011229158 (*Thermus thermophilus* HB8), 2PZ0_A (*Thermoanaerobacter tengcongensis*), WP_011250348 (*Thermococcus kodakarensis* KOD1) and AAL82127.1 (*Pyrococcus furiosus* DSM 3638).

The *pfGDPD* gene is 762 bp in length, encoding a 253 aa protein (Figure 1A). No potential transmembrane domains and signal peptide region was found by analysis of protein sequence, which indicated it is a cytoplasmic protein in the cell. A highly conserved sequence motif (HR(X)*^n^*EN (X)*^n^*EXD(X)*^n^*HD) comprising the active site residues was found in the pfGDPD enzyme, with histidine 17 and histidine 59 as the putative catalytic residues (Figure 1B). pfGDPD exhibited highly sequence identity with the GDPD from *Thermococcus kodakarensis* KOD1(PDB:4OEC) (77%). It shares 41% and 35% sequence identity with GDPD from *Thermotoga maritima* (PDB:1O1Z) and GDPD from *Thermoanaerobacter tengcongensis* (PDB:2PZ0) (Figure 1B), respectively. In addition, the divalent cation-binding related residues in that cleft of GDPDs (Figure 1B) were also seen in all the four GDPDs, which indicates its evolutionary conservatism. Results of phylogenetic analysis indicates that the pfGDPD has a very high relationship with GDPD from *Thermococcus kodakarensis* KOD1 (Figure 2). The very high level of sequence identity, conserved sequence motif and the high phylogenetic relationship provide strong evidence that this molecule is a member of the GDPD superfamily.

### 2.2. Expression and Purification of the Recombinant pfGDPD

**Figure 3:**
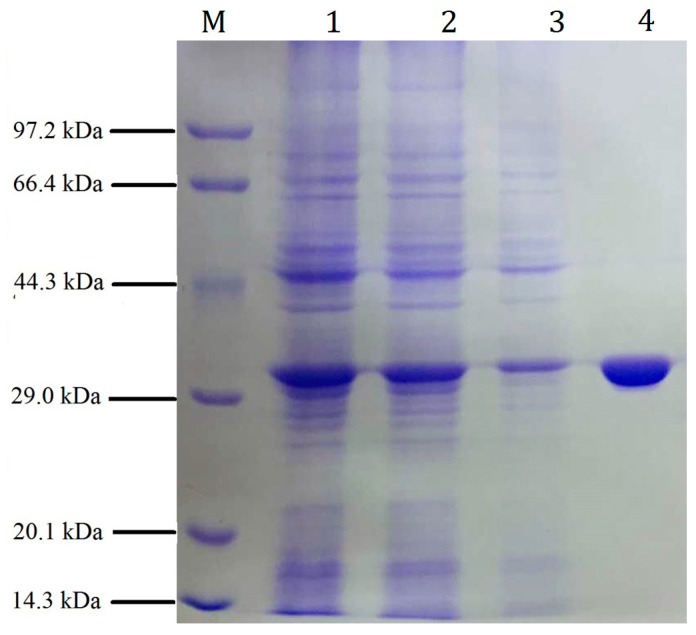
Sodium dodecyl sulphate-polyacrylamide gel electrophoresis (SDS-PAGE) analysis of total expressed cell proteins from *E. coli* (harboring pET28a-(His)6-pfGDPD-(His)6) vector and the eluted fractions from nickel-chelate chromatography. **Lanes M**: Molecular marker; **Lanes 1**: Total cell lysate; **Lanes 2**: Supernatant of total cell lysate that centrifugated with 11,000× *g* for 10 min; **Lanes 3**: Precipitation of total cell lysate that centrifugated with 11,000× *g* for 10 min; **Lanes 4**: Nickel-chelate chromatography and eluted with washing buffer that contained 200 mM imidazole.

The *pfGDPD* gene fusing both N- and C-terminal his-tags was cloned into a pET28a vector and then expressed in SHuffle T7 Express Competent *E. coli* strain. Sodium dodecyl sulphate-polyacrylamide gel electrophoresis (SDS-PAGE) analysis results indicated that the recombinant protein (pfGDPD) was soluble in the supernatant of the cell lysate and was beneficial for further purification (Figure 3). The pfGDPD was further purified with nickel-chelate chromatography to get a single band (Figure 3). The precise molecular weight of recombinant phGDPD was found to be 34,059 Da (Figure S1), and the purified recombinant protein was further verified by liquid chromatograph-mass spectrometer (LC-MS) to be the protein that we want to characterize (Figure S2).

### 2.3. Biochemical Characterization of Recombinant Enzyme

#### 2.3.1. Effect of Temperature on Enzymatic Activity and Thermostability of Recombinant pfGDPD

**Figure 4:**
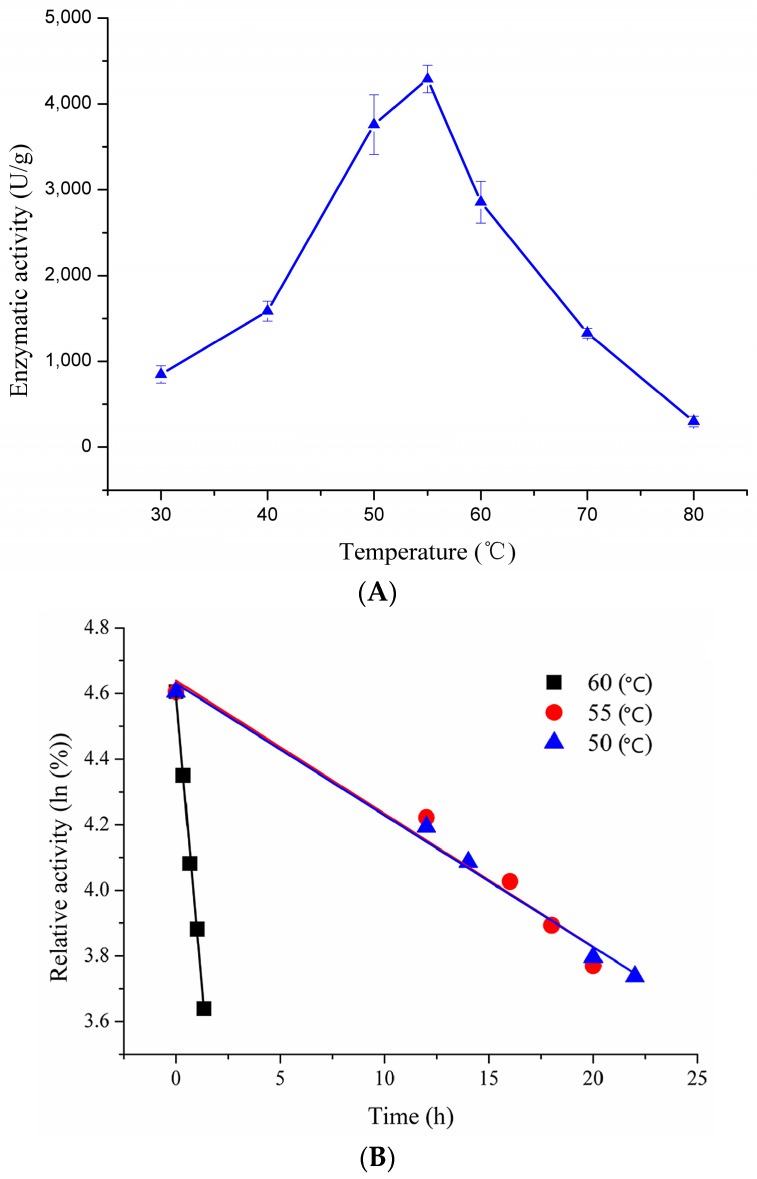
Effect of temperature on activity (**A**) and thermostability (**B**) of pfGDPD. (**A**) The purified pfGDPD was assayed at different temperatures (30–80 °C). Enzymatic activities were tested with bis(para-nitrophenol) phosphate (BpNPP) as substrate. Values are means ± standard deviation from three independent experiments; (**B**) The enzymatic activity was assayed after incubation in different temperatures (50, 55 and 60 °C). Activity was displayed as percentages of the initial activity. The data were fitted to the first-order plots. The half-lives of pfGDPD were calculated as 17.6 h, 17.8 h and 56 min, respectively.

The optimal temperature of recombinant pfGDPD was determined using BpNPP as substrate. Recombinant pfGDPD showed highest activity at 55 °C (Figure 4A). However, the enzymatic activity declined quickly when the temperature was raised above 60 °C or reduced below 40 °C, increasing the temperature to 80 °C resulted in almost complete loss of enzymatic activity. Thermostability of recombinant pfGDPD was investigated at three different temperatures (50, 55 and 60 °C) with increasing incubation time (Figure 4B). The half-lives of pfGDPD were calculated as 17.6 h, 17.8 h and 56 min, respectively. At 45 °C, the half-life of pfGDPD was six days. Results of circular dichroism spectral analysis on thermal unfolding of pfGDPD indicated that the secondary structure of pfGDPD remained stable even at 55 °C; however, further increasing the temperature caused a change in the spectra, especially for the peak of 192 nm (Figure S3). The *T*_m_ value of pfGDPD was calculated to be 59.6 °C, which coincided with the results of thermostability.

#### 2.3.2. Effect of pH on Enzymatic Activity of Recombined pfGDPD

**Figure 5:**
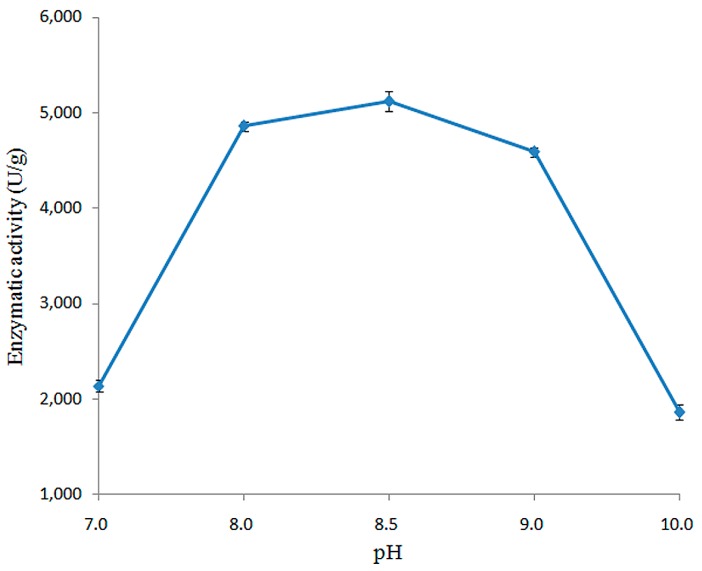
Effect of pH on activity of the purified pfGDPD. Enzymatic activities were measured under various pH buffers under standard assay conditions with BpNPP as substrate.

The activity of recombined pfGDPD was measured over a pH range from 4.0 to 10.0. As shown in Figure 5, the enzyme was only active within a narrow pH range (7.0 to 10.0), with optimum activity at pH 8.5. No activity was detected when the pH value lower than 7.0 (Figure 5).

#### 2.3.3. Effects of Metal Cations on Enzymatic Activity of Recombinant pfGDPD

To determine the effect of metal ions on pfGDPD activity, enzyme activities were tested in the presence of various metal ions. As can be seen from Table 1, no activity was found when no metal cations added or ethylene diamine tetraacetie acid (EDTA) was added. Among various metal cations studied here, except Ca^2+^, Li^2+^, Fe^3+^ and Zn^2+^, all other cations tested here had stimulatory effects on the catalytic activity of recombinant pfGDPD. Enzymatic activity of pfGDPD was strongly activated by Mn^2+^ at the concentration of 5.0 × 10^−3^ M. Higher concentrations in the reaction system may cause the denaturing of protein and reduce the activity further.

#### 2.3.4. Effect of Organic Solvents on Recombined pfGDPD Activity

**Figure 6:**
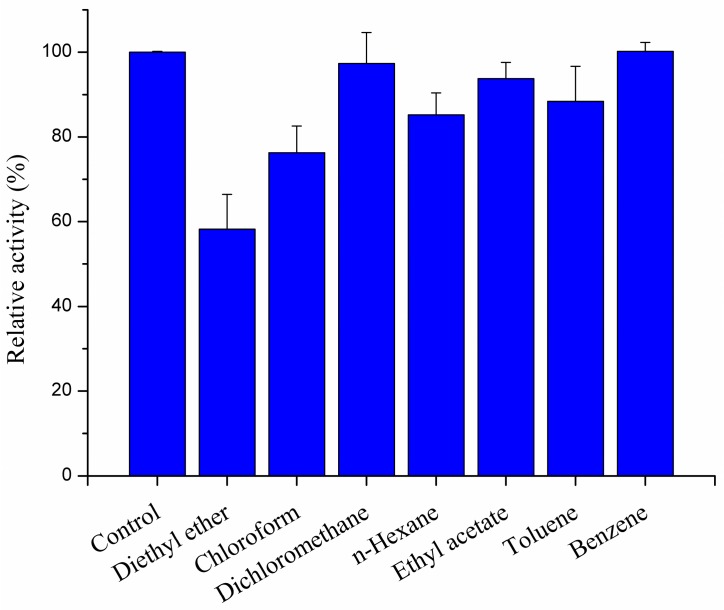
Effect of different organic solvents on pfGDPD activity. The enzyme solution was incubated separately with each organic solvent (final concentration 50%, *v*/*v*) at 4 °C for 1 h, and then the residual activity of enzyme was measured at 55 °C and pH 8.5 using BpNPP as substrate.

The influence of various organic solvents on the activity of recombined pfGDPD was also investigated (Figure 6). The enzyme activity was inhibited with diethyl ether by 59% of its initial activity. It was not affected by dichloromethane and benzene and was weakly inhibited by chloroform, *n*-hexane, ethyl acetate and toluene.

### 2.4. Hydrolytic Reaction

**Figure 7:**
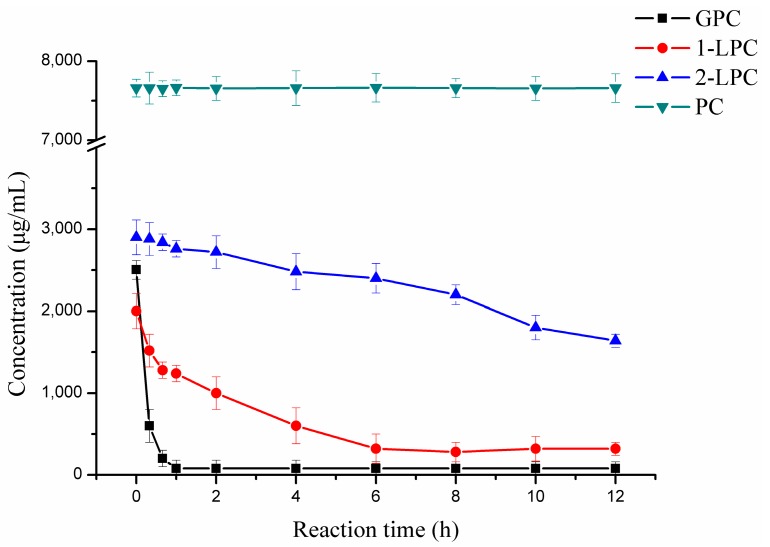
The hydrolysis process curve of l-α-glycerophosphorylcholine (GPC), *sn*1-l-α-lysophosphatidylcholine (1-LPC), *sn*2-l-α-lysophosphatidylcholine (2-LPC), and l-α-phosphatidylcholine PC by pfGDPD. Conditions: GPC, LPC or PC (24.3 mM), pfGDPD (25 U), 1 mL buffer (50 mM Tris-HCl, pH 8.5), temperature 45 °C and reaction time 12 h.

In the present study, in order to verify whether the enzyme could use l-α-lysophosphatidylcholine (LPC) or l-α-phosphatidylcholine (PC) as hydrolytic substrate, the hydrolytic activity of pfGDPD toward various phospholipids, l-α-glycerophosphorylcholine (GPC) LPC and PC, was tested. There was a sharp decrease in GPC concentration in the reaction mixture within 1 h of reaction (Figure 7), with the concentration of GPC decreasing from 2504 μg/mL to be only 81 μg/mL. Compared with 2-LPC, there was a great decrease in the concentration of 1-LPC. After 6 h of reaction, the concentration of 1-LPC and 2-LPC decreased from 2000 and 2900 μg/mL, respectively, to be 600 and 2480 μg/mL, respectively indicating that pfGDPD preferred 1-LPC to 2-LPC. The concentration of PC was not significantly different within the 12 h reaction (Figure 7).

### 2.5. Molecular 3D-Model of pfGDPD and Docking of GPC, 1-LPC, 2-LPC to pfGDPD

**Figure 8:**
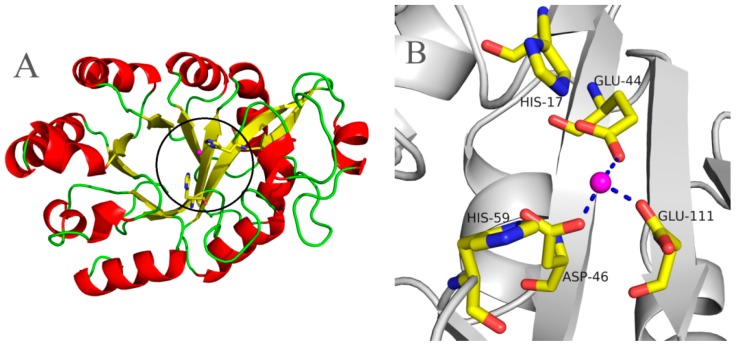
Molecular 3D model of pfGDPD and locations of the deduced catalytic sites. (**A**) the 3D model of pfGDPD protein was built with a SWISS-MODEL Web Server (Basel, Switzerland) by using the crystal structure of *Thermococcus kodakarensis* KOD1 GDPD (PDB: 4OEC) as template. The colored segments of the backbone structure mark the location of α helix (red), β sheet (yellow) and loop (green). The black circle shows the active region; (**B**) the active site of two histidine residues are shown as a stick. A bivalent cation is shown in magenta, and broken blue lines represent the hydrogen bonds that form with Glu44, Asp46 and Glu111 residues.

Using the crystal structure of GDPD from *Thermococcus kodakarensis* KOD1 (PDB: 4OEC) as the model, a 3D-model of pfGDPD was constructed. Similar to other GDPDs, the pfGDPD monomer exhibited a triosephosphate isomerase (TIM)-barrel fold and displayed a central eight-stranded (β1, β2, β4, β5, β6, β7, β8, β9) parallel β-sheet barrel, which was surrounded by ten α-helices (α1, α3, α4, α5, α6, α7, α8, α9, α10, α11) (Figure 8A). According to the structure of 4OEC, Glu44, Asp46, and Glu109 constituted a metal-binding site in that cleft of GDPD, with an Mg atom bound (Figure 8B).

**Figure 9:**
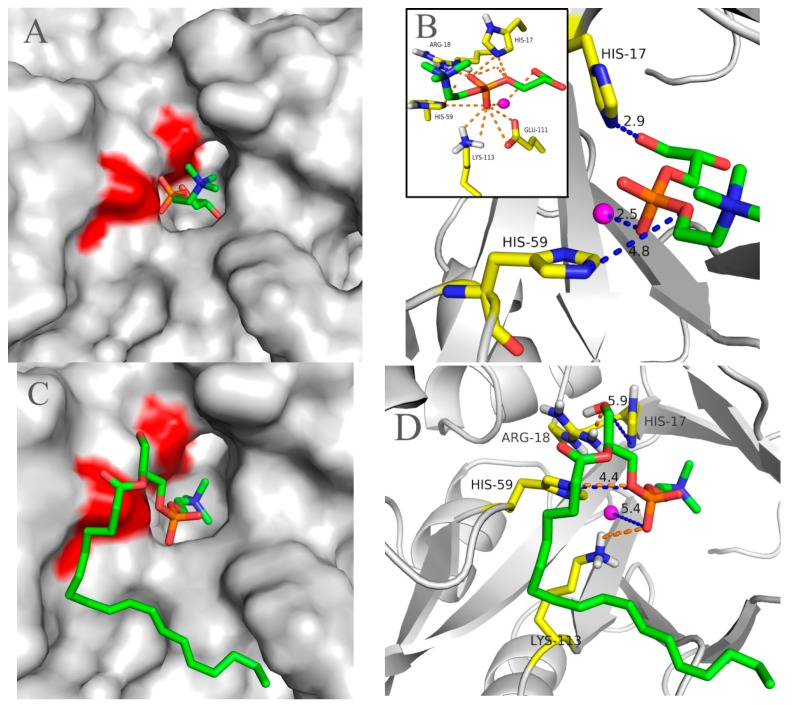

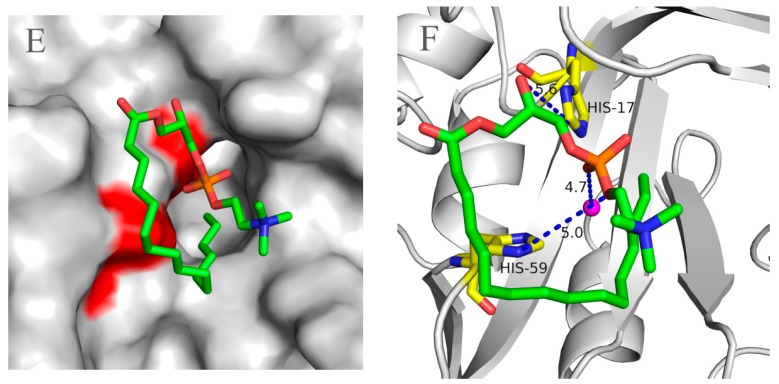
The 3D structures of the pfGDPD-phospholipid complexes. (**A**) surface view of the most suitable binding position and orientation of GPC. Two catalytic histidine residues are shown in red; (**B**) a close-up view showing the distance between the GPC and two histidine residues as well as the bivalent cation (shown in magenta). The hydrogen bonds formed with related residues (in the **black** pane); (**C**) surface view of the most suitable binding position and orientation of 1-LPC. Two catalytic histidine residues are shown in red; (**D**) a close-up view showing the distance between the 1-LPC and catalytic two histidine residues as well as the bivalent cation (shown in **magenta**) by dashed line in blue. The hydrogen bonds formed with related residues aremarked by dashed line in orange; (**E**) surface view of the most suitable binding position and orientation of 2-LPC. Two catalytic histidine residues are shown in red; (**F**) A close-up view showing the distance between the 2-LPC and catalytic two histidine residues as well as the bivalent cation (shown in **magenta**) by dashed line in blue.

To understand the structural foundation for pfGDPD to hydrolyze GPC, 1-LPC and 2-LPC, these three substrates were docked into the catalytic pocket of pfGDPD. As shown in Figure 9A, GPC was easily docked into the catalytic pocket of pfGDPD. The distance between OH2 group of GPC and NE2 of His17 was 3.2 Å (Figure 9B). NE2 of His59 and the O3 on phosphorylcholine showed a distance of 4.0 Å, while the distance between the divalent cations and O4 on the phosphorylcholine was 3.6 Å. In addition, a close hydrogen bonding network of GPC with His17, Arg18, His59, Glu111 and Lys113 was also found (Figure 9B). In the structure of pfGDPD with 1-LPC or 2-LPC complex (Figure 9C,E), due to the space limitation in the binding pocket that does not have enough space to accommodate the fatty acid chains inserted into the narrow pocket, accordingly, the fatty acid chains of 1-LPC and 2-LPC only laid in the groove outside the binding pocket and the sn-3 phosphatidyl choline near the leaving channel. The distance between the OH1 group of 1-LPC and NE2 of His17 was 5.9 Å. The NE2 of His59 and the O3 on phosphatidyl showed a distance of 4.4 Å, while the distance between the divalent cations and O4 on the phosphatidyl was 5.4 Å (Figure 9D). Only three pairs of hydrogen bonds were found between 1-LPC and Arg18, His 59, Lys113, respectively (Figure 9D). The distance between OH2 group of 2-LPC and NE2 of His17 was 5.6 Å. The NE2 of His59 and the O3 on phosphatidyl showed a distance of 5.0 Å, while the distance between the divalent cations and O4 on the phosphatidyl was 4.7 Å (Figure 9F). In the present study, we failed to dock PC into the catalytic region of pfGDPD. These data are consistent with our experimental results that pfGDPD could hydrolyze GPC and LPC, but not PC.

## 3. Discussion

During enzymatic characterization of GDPDs, two methods have been used for the activity testing. One method determined glycerol-3-phosphate production in a coupled spectrophotometric assay, with glycerophosphocholine, glycerophosphoethanolamine, glycerophosphoglycerol, glycerophosphoinositol, or glycerophosphoserine as substrates. It was found that GDPDs had activity in these substrates, and showed no selectivity in terms of the alcohol moiety [3]. In the present study, we also used this method to test the hydrolytic activity of pfGDPD towards GPC. The activity was 868.72 U/mg under optimal conditions. However, the high price of the substrate and related enzymes, and time-consuming assay, restricted the application of this method for large scale work. Moreover, along with the finding that GpdQ from *Enterobacter aerogenes* had potential utility in OP bioremediation, much attention has been given to evaluate the hydrolytic activity of GpdQ to these substrates [15,16]. Since most OP compounds are toxic and should be used with the appropriate safeguards, some mimic substrates of OP that carry the para-nitrophenol group were used for the testing of hydrolytic activity to high throughout screen of the enzyme that could be used in bioremediation [14]. Among these, bis(para-nitrophenol) phosphate (BpNPP), was one kind of substrate that was easily obtained and most frequently used to assay phosphodiesterases [15,16]. In the present study, in order to investigate the potential of pfGDPD in the degradation of OP, enzymatic characterization of this enzyme was conducted with this method. We found that the pfGDPD could use this substrate, which indicated that the enzyme may have potency to be used in the monitoring or degrading of OP esters. Further research on this application of this enzyme is underway. The present results provide the basic biochemical information on the enzyme and facilitate our further application research.

Structurally, GDPD is a metallophosphoesterase of the α/β sandwich structural fold [17]. The structures of several GDPD from prokaryotes have been determined. It has been shown that a Ca atom or some similar metal ion (Mg, Zn, Fe, *etc.*) is found at the active site [18]; such enzymestypically have particular metal-ion speficities, which are usually reflected in their physiological function. Therefore, the characterization of the native metal ion preference of GDPD was an important step towards understanding its physiological function and catalytic mechanism. The present results indicated that Mn^2+^ ions were the most active on the activity of pfGDPD. However, different from other reported GDPDs, no stimulatory effect on the catalytic activity was found for Ca^2+^, Fe^3+^ or Zn^2+^ whichfurther indicates its novelty. In addition, three hydrogen bonds were formed with Glu44, Asp46 and Glu111 residues to the bivalent cation (Figure 8B), which were coincident with the structure of ttGDPD. These three residues were highly conserved between different GDPDs (Figure 1). It had been found that mutation of ttGDPD for any of these three residues to alanine inactivates the enzyme [18]. Furthermore, polar contact of metal with GPC and 1-LPC were both found from the docking results (Figure 9B,D). The role of the metal in the hydrolytic reaction may be acting as an electrophile to stabilize the intermediate formed during the first step of the reaction [18].

Structurally, GDPDs form a large family with an evolutionarily conserved sequence motif (HR(X)*^n^*EN (X)*^n^*EXD(X)*^n^*HD) comprising the active site residues, which is shared by the catalytic domain of the phosphoinositide-specific phospholipase C (PI-PLC) family [19,20,21]. In the pfGDPD, the conserved sequence motif was also found and His17 and His59 were strictly conserved in the cleft of GDPD, and mainly function as a mechanism of general base and acid which participate in the catalytic process [18]. Due to the high identity of pfGDPD and ttGDPD mentioned above, a similar catalytic mechanism may exist in the pfGDPD. Docking of GPC to the catalytic pocket of pfGDPD was successful with the choline group toward the outside and glycerol moiety toward the pocket. This orientation was similar with the modeled results obtained by GpdQ, with the orientation of the natural substrate glycerophosphoethanolamine (GPE) [22] and further indicated the rationality of GPC in the catalytic pocked of pfGDPD. The outside phosphate moiety in the leaving channel may facilitate the releasing of choline from the catalytic pocket and inside glycerol moiety in the binding pocket may make it feasible for the His17 to accept a proton from the OH group of the glycerol moiety of glycerophosphodiester. To date, two members of GDPDs from mammalian, GDE4 and GDE7, have been reported to show lysophospholipase D activity; however, no activity was found for glycerophosphodiesters [23]. In addition, purified GDE4 was found to show lyso-PLD activity toward *N*-acylethanolamine lysophospholipids, lysophosphatidylethanolamine and lysophosphatidylcholine [24]. In the present study, we found that, except for GPC, pfGDPD could catalyse the hydrolysis of 1-LPC and 2-LPC, which indicated that the enzyme was special in its substrate selectivity. Docking results indicated that the close distance between these atoms and powerful hydrogen bonding network in common made for efficient enzyme catalysis GPC. In contrast, only three hydrogen bond were found to stabilize 1-LPC in the catalytic pocket of pfGDPD, the deduced hydrogen bond and larger distance between 1-LPC and two catalytic histidine residues (Figure 9D), possibly the reason why lower activity was observed in the hydrolytic reaction for 1-LPC than GPC. For the 2-LPC, although the distance between the substrate and catalytic residues (His17, His59) was similar, no hydrogen bond existed in the corresponding site with residues mentioned before (His17, Arg18, His59, Glu111 and Lys113). This docking result indicated that 2-LPC may not be stable in the catalytic pocket, and further induced the low activity of pfGDPD to 2-LPC. In the present study, docking of PC to the catalytic pocket of pfGDPD failed. It may be the stereospecific blockade in the catalytic pocket that prevents both the sn-1 and sn-2 moiety of PC entering the pokey catalytic pocket and inducing no activity towards PC. Similar results were also found for mammalian GDE4 and GDE7 which did not hydrolyze 1,2-dibutyrylsn-glycero-3-phosphatidylcholine [23].

## 4. Materials and Methods

### 4.1. Chemicals, Enzymes and Strains

*Escherichia coli* DH5α, kanamycin, restriction endonucleases (*EcoR*I and *Xho*I) and Isopropyl β-d-1-thiogalactopyranoside (IPTG) were purchased from Takara Co., Ltd. (Dalian, China). SHuffle T7 Express Competent *E. coli* was purchased from New England BioLabs (Beijing, China). pET28a expression vector was purchased from Stratagene (La Jolla, CA, USA). Ni^2+^-nitrilotriacetate (Ni^2+^-NTA) resin was obtained from Qiagen Inc., (Valencia, CA, USA). The hydrolytic substrate bis(*p*-nitrophenyl) phosphate sodium salt (BpNPP), l-α-glycerophosphorylcholine (GPC), l-α-lysophosphatidylcholine (LPC) and l-α-phosphatidylcholine (PC) used in the present study were all purchased from Sigma-Aldrich (Sigma Chemical Co., St. Louis, MO, USA). All other chemicals used in the present study were of analytical grade.

### 4.2. Sequence Analysis of pfGDPD

The pfGDPD protein from *Pyrococcus furiosus* DSM 3638 that expressed in this study was deposited in the NCBI-Proteindatabases under the accession number of AAL82127.1. The potential transmembrane domains of pfGDPD were predicted by TMHMM2.0 server ([25]. The molecular mass and isoelectric point of the deduced pfGDPD protein were predicted using the Compute pI/*M*_w_ tool at the ExPASy molecular biology web server of the Swiss Institute of Bioinformatics (http://www.expasy.org/) [26]. The deduced signal peptide was predicted using SignalP 4.1 server [27]. Similarity searches were performed with the BLAST 2.0 program (Bethesda, MD, USA) [28]. Multiple sequence alignments were performed with ClustalW2 (Belfield, Dublin, Ireland) [29]. Phylogenetic analysis with Neighbor-joining (NJ) was conducted using MEGA 6.0 software (Hachioji, Tokyo, Japan) [30].

### 4.3. Expression and Purification of Recombinant pfGDPD

The pfGDPD gene that harbored a restriction endonucleases site of *Eco*RI and *Xho*I was artificially synthesized by Sangon Biotech, Inc. (Shanghai, China). The gene sequence was just based on the sequence provided on *Pyrococcus furiosus* DSM 3638, complete genome (GenBank accession number: AE009950.1) complement 11852111-1852872 (GI: 18894211). Information on the synthetic sequence was listed in Figure 1A. The gene encoding the peptide was cloned into pET28a vector that contained 6× his-tag to form pET28a (His)_6_-pfGDPD-(His)_6_ and transformed into *E. coli* DH5α. Plasmids were confirmed by sequencing. The constructed vector was further transformed into Shuffle T7 Express Competent *E. coli*. To purify pfGDPD, Shuffle T7 Express Competent *E. coli* cells that harbor pET28a-pfGDPD were grown at 37 °C in 1 L of LB medium that contained 2 mL of 1.0 M kanamycin, and induced at an optical density of 0.8 at 600 nm by IPTG to a final concentration of 0.05 mM. After 12 h of induction at 20 °C, the cells were harvested, re-suspended in 100 mL of 20 mM Tris-HCl (pH 8.0) and disrupted by sonication (ULTRASONIC PROCESSOR UH-950A, Tianjin Automatic Science Instrument Co. Ltd., Tianjin, China). The cell lysate was then centrifuged at 11,000× *g* for 10 min to remove insoluble cell debris, and the supernatant was assayed before further purification.

To further purify pfGDPD, the supernatant was then filtered through a 0.45 μm filter and applied to a Ni^2+^-NTA-agarose column (bed volume 40 mL). The target enzyme was eluted with 20 mM Tris-HCl buffer (pH 8.0) that containing 200 mM imidazole. The enzyme-containing eluent was further filtered though a Sephadex G-25 column (GE Healthcare, Amersham Biosciences UK Ltd., Buckinghamshire, UK) to remove the imidazole. The fraction containing purified enzymes were collected and analyzed by 12% SDS-PAGE. Protein concentrations were determined by the BCA Protein Assay Kit (Sangon Biotech, Shanghai Co., Ltd., Shanghai, China).

### 4.4. Biochemical Characterization of Recombinant Enzyme

#### 4.4.1. Enzyme Activity Testing

The enzymatic activity was determined by colorimetric method using BpNPP as substrate according to the method reported before [15]. One unit of enzyme activity is defined as the amount of enzyme required to release 1 nmol of *p*-nitrophenol per minute. The hydrolytic activity to GPC was determined by measuring the amount of choline released from the substrate GPC according to the method of Liu *et al.* [31] with minor modifications by using GPC instead of PC. One unit (U) of hydrolytic activity of pfGDPD was defined as the amount of enzyme that produced 1 μmol of choline per minute under optimal conditions.

#### 4.4.2. Determining Temperature Optimum for the Activity and Thermostability of pfGDPD

The optimum temperature of the pfGDPD was evaluated at pH 8.5 using BpNPP as substrate. The temperature were set from 30 to 80 °C. Thermostability of pfGDPD was tested by pre-incubating the pfGDPDat different temperatures. Samples were then taken at different intervals for measurement of residual activity under the above assay conditions (50 mM Tris-HCl, pH 8.5, optimum temperature). The temperatures were set as 45, 50, 55 and 65 °C, respectively. Circular dichroism spectral analysis on thermal unfolding of pfGDPD in Tris-HCl buffer (5 mM, pH 8.5) was monitored by a chrascan spectropolarimeter (Applied Photophysics, Surrey, UK) according to the method reported by Lan *et al.* [32]. The *T_m_* values were calculated from the spectra using Global 3™ Analysis software (Applied Photophysics).

#### 4.4.3. Determining pH Optimum for the Activity of pfGDPD

Optimum pH value for the pfGDPD was determined at 55 °C using BpNPP as substrate. The buffers used in this study included 50 mM citric acid-sodium citrate buffer (pH 4.0 and 5.0), 50 mM phosphate buffer (pH 6.0 and 7.0), 50 mM Tris-HCl (pH 8.0), 50 mM Tris-HCl (pH 8.5) and 50 mM Gly-NaOH (pH 9.0).

#### 4.4.4. Effect of Metal Ions on the Enzymatic Activity of pfGDPD

The influence of metal ions on the activity of pfGDPD was determined in the presence of various metal ions. Enzymatic activity was tested at 55 °C and 50 mM Tris-HCl, pH 8.5 using BpNPP as substrate. Metal ions including Mn^2+^, Co^2+^, Ni^2+^, Cu^2+^, Zn^2+^, Mg^2+^, Fe^3+^, Ca^2+^ and Li^2+^ at a final different concentration, varied from 1.0 to 100 mM, were used in this study. The effect of ethylenediaminetetraacetic acid (EDTA) at 10 mM concentration on enzyme activity was also investigated as described above.

#### 4.4.5. Effect of Organic Solvents on the Enzymatic Activity of pfGDPD

The effect of various organic solvents (Diethyl ether, Chloroform, Dichloromethane, *n*-Hexane, Ethyl acetate, Toluene, Benzene) on pfGDPD activity was determined. The enzyme solution was incubated separately with each organic solvent (final concentration 50%, *v*/*v*) at 4 °C for 1 h, and then the residual activity of enzyme was measured at 55 °C and pH 8.5 using BpNPP as substrate.

### 4.5. Hydrolytic Reaction and Analysis

A reaction mixture, containing either PC, LPC or GPC with the same concentration (24.3 mM), and 25 U pfGDPD (tested with GPC as hydrolytic substrate), and 1.0 mL buffer (50 mM Tris-HCl, pH 8.5) was used to investigate the hydrolytic ability of pfGDPD with different phospholipids. Reactions were carried out in a thermomixer with a speed of 200 rpm at 45 °C for 24 h. In addition, a 40 μL sample was withdrawn periodically during the 24 h of reaction. Samples (40 μL) withdrawn from the reaction mixture, were diluted with 960 μL methanol, mixed and centrifuged at 10,000× *g* for 2 min. The supernatant was filtered through a Millipore membrane (0.22 μm, from Roth) and analyzed by HPLC. Analysis of the PC, 1-LPC, 2-LPC and GPC in hydrolysates was performed on an HPLC (Waters-1525, Milford, MA, USA) with an ELSD (Deerfield, IL, USA) and a Symmetry C18 column (4.6 mm × 150 mm, 5 mm, Wasters) according to the method reported by Wang *et al.* [33]. PC, 1-LPC, 2-LPC and GPC were identified and quantified by comparison with peak retention times and calibration curves of standards.

### 4.6. Molecular Modeling of pfGDPD and Molecular Docking Simulation

The 3D model of the pfGDPD protein was built with SWISS-MODEL Web Server (http://swissmodel.expasy.org/) [34] (Basel, Switzerland) using the crystal structure of *Thermococcus kodakarensis* KOD1 GDPD (PDB accession code 4OEC) as template. Libdock (Discovery Studio, Accelrys lnc., San Diego, CA, USA) was employed to analyze the interaction of pfGDPD with GPC 1-LPC, 2-LPC and PC. The complex structure that had the most suitable binding position and orientation was selected to be analyzed. The modeled 3D structure and modeled complex structures were visualized and analyzed using the PyMOL software (Palo Alto, CA, USA) [35].

### 4.7. Statistical Analysis

All experiments were performed in triplicate. Results are reported as means ± standard deviations (SD) of these measurements. All curves were fitted using Microsoft Office Excel 2007 (Microsoft Corporation, Redmond, WA, USA) and Origin 8.0 (OriginLab Corporation, Northampton, MA, USA).

## 5. Conclusions

In summary, we have biochemically characterized a novel GDPD from different angles. A very interesting finding was that the enzymes possess lysophospholipase D activity and have the potential to be used in the monitoring or degrading of organophosphorus (OP) esters. Considering the great hazard that OP esters have made to humans and the environment, and the great demand for enzymatic degradation, further studies will concentrate on the evaluation and further improvement of this enzyme for bioremediation applications.

## Figures and Tables

**Table 1:** Effect of metal ions on the activity of purified pfGDPD.

Ions	Concentration (mM)	Enzymatic Activity (U/g)
None	—	N.D.
EDTA	10	N.D.
Mn^2+^	1	4515 ± 187
	5	5842 ± 6
	10	5677 ± 51
	50	2864 ± 129
	100	1967 ± 266
Co^2+^	1	4972 ± 58
	5	3935 ± 138
	10	2903 ± 133
	50	1536 ± 229
	100	1128 ± 179
Ni^2+^	1	1041 ± 42
	5	1893 ± 54
	10	1723 ± 90
	50	719 ± 93
	100	359 ± 69
Cu^2+^	10	370 ± 202
Mg^2+^	10	74 ± 19
Ca^2+^	10	N.D.
Li^+^	10	N.D.
Fe^3+^	10	N.D.
Zn^2+^	10	N.D.

Note: N.D. represent not detected.

## Supplementary Materials

Supplementary materials can be found at http://www.mdpi.com/1422-0067/17/6/831/s1.
