# Supplementary material for: Expression and Characterization of a Novel Glycerophosphodiester Phosphodiesterase from Pyrococcus furiosus DSM 3638 That Possesses Lysophospholipase D Activity
Source: Int J Mol Sci. 2016 May 30;17(6):831. doi: 10.3390/ijms17060831 (PMC4926365; doi:10.3390/ijms17060831)
Supplement: Supplementary file 1 [file ijms-17-00831-s001.pdf]

Supplementary Materials: Expression and Characterization of a Novel Glycerophosphodiester Phosphodiesterase from *Pyrococcus furiosus* DSM 3638 That Possesses Lysophospholipase D Activity

Fanghua Wang, Linhui Lai, Yanhua Liu, Bo Yang and Yonghua Wang

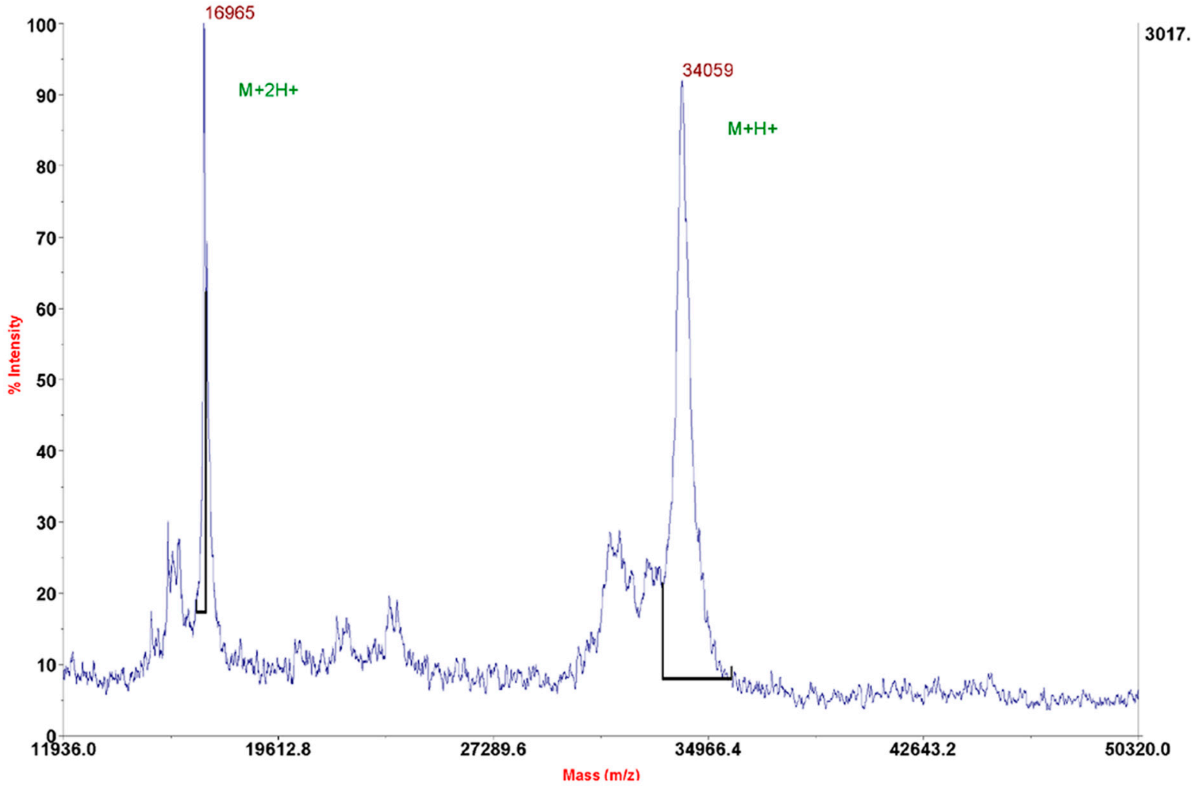

Figure S1. MALDI-TOF-MS analysis on the detail molecular weight of recombinant pfGDPD. Purified pfGDPD was analyzed by MALDI-TOF/TOF 5800 (AB SCIEX, USA) with lineral mode, positive detector.

1. tr|Q8TZI9|Q8TZI9_PYRFU Glycerophosphodiester phosphodiesterase OS=Pyrococcusfuriosus (strain ATCC 43587 / DSM 3638 / JCM 8422 / Vc1) GN=PF2003 unused score=108.17,coverage=92.9%;

MVGNPMWERDKIIVLGHRGYMAKYPENSLLSIRKAIEAGADGVEIDVWLSKDNKVILMHDETIDRT
SNLKGRQKEMTLEELKKANIGMGERIPTLEEVFEILPKDALLNIEIKDRDAAKEVARIVSENNPER
VMISSFDIEALREYRKYDDTTIMGLLVDKEETVPLIPKLKEKLNLWSVNVPMEAIPIIGFEKTYQA
IKWVRSLGLKIVLWTEDDKLFYVDENLKRLLGMFEVVIANDVERMVSYLSSLGIR

Note: The green part represents the mass spectrometry identified peptide segment sequence with more than 95% of the credibility.

Partial peptide mass spectrometry：

AIEAGADGVEIDVWLSK

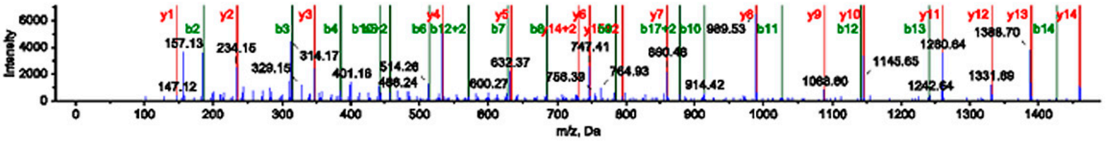

AIEAGADGVEIDVWLSKDNK

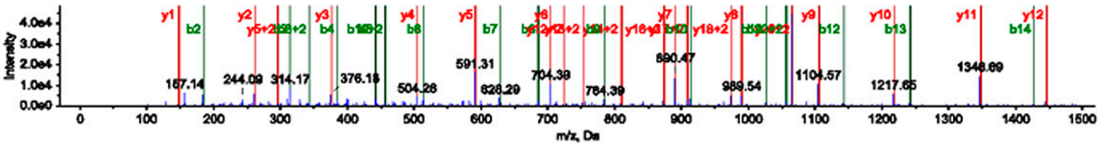

AIEAGADGVEIDVWLSKDNKVILMHDETIDR

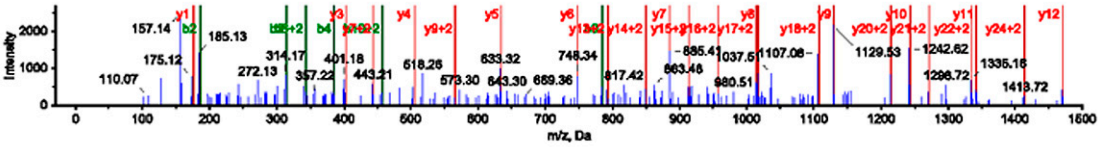

ANIGMGER

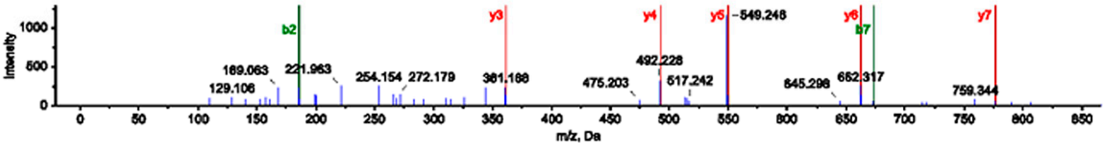

ANIGMGERIPTLEEVFEILPK

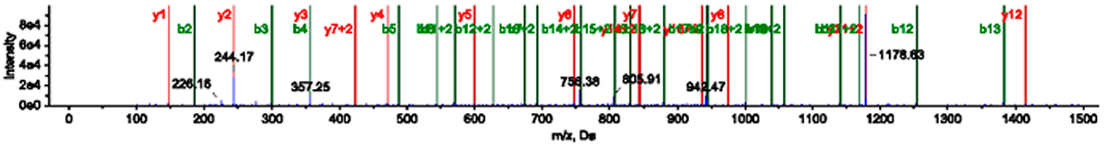

DALLNIEIK

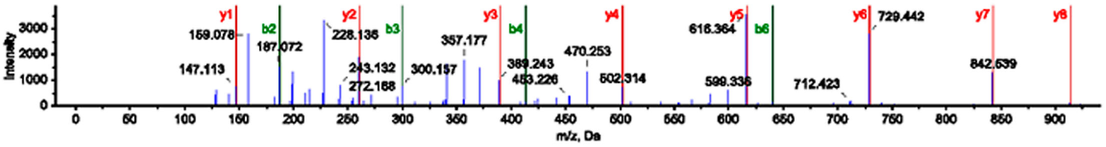

Model：Triple TOF 5600 plus LCMS (AB SCIEX USA）

Figure S2. LC-MS results of purified pfGDPD. The target band in the SDS-PAGE was cut, enzymatic digested and tested by Triple TOF 5600 plus LCMS (AB SCIEX USA).

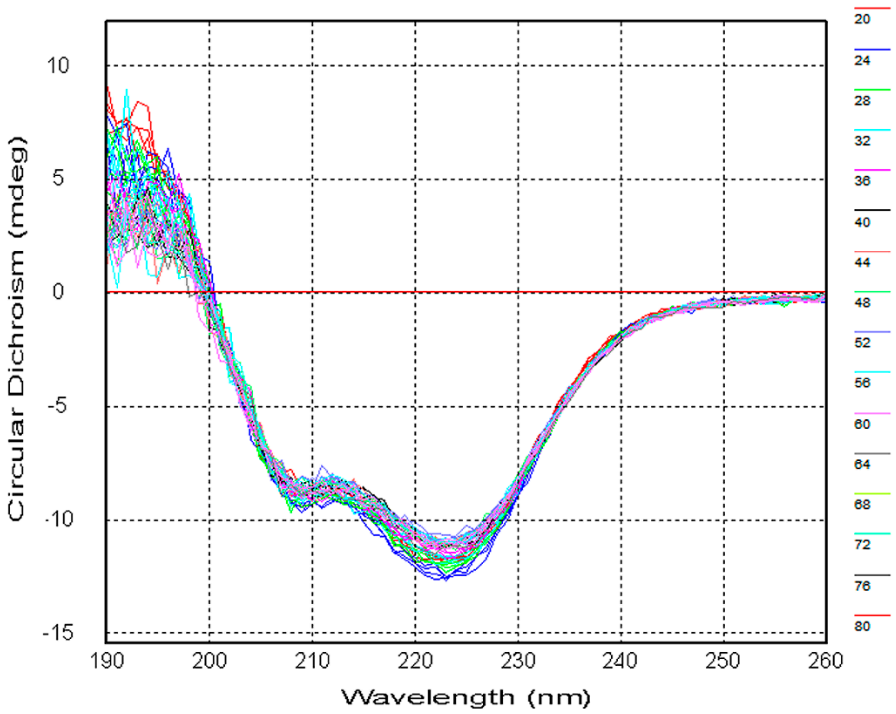

Figure S3. Circular dichroism spectra of recombinant pfGDPD under different temperature. CD spectral of recombinant pfGDPD in Tris-HCl buffer (5 mM, pH 8.5) were monitored by a chrascan spectropolarimeter (Applied Photophysics, Surrey, UK). The spectra were recorded over a wavelength range of 190–260 nm using a 1-mm cuvette at a scan speed of 100 nm per min and a response time of 1 s. The temperature was increased at a heating rate of 2 °C per minute from 20 to 90 °C.
